# Reproduction of *Meloidogyne enterolobii* on selected root-knot nematode resistant sweetpotato (*Ipomoea batatas*) cultivars

**DOI:** 10.21307/jofnem-2020-063

**Published:** 2020-07-21

**Authors:** Janete A. Brito, Johan Desaeger, D.W. Dickson

**Affiliations:** 1 Florida Department of Agriculture and Consumer Services, Division of Plant Industry, Gainesville, FL 32614-7100; 2 Entomology and Nematology Department, Gulf Coast Research and Education Center, University of Florida, Wimauma, FL 33598; 3 Entomology and Nematology Department, Institute of Food and Agricultural Sciences, University of Florida, Gainesville, FL 32611-0620

**Keywords:** Guava root-knot nematode, *Meloidogyne enterolobii*, Pacara earpod tree root-knot nematode, Reproduction, Root-knot nematode, Susceptibility, Sweetpotato

## Abstract

The ability of *Meloidogyne enterolobii* to reproduce on selected sweetpotato (*Ipomoea batatas*) cultivars (Beauregard, Covington, Evangeline, Hernandez, and Orleans (LA 05-111)) was evaluated in two greenhouse experiments, each with 10 replicates. All cultivars, except Beauregard (control) and Orleans, were reported previously as moderately resistant or resistant to *M. incognita, Fusarium oxysporum* f. sp. *batatas*, and *Streptomyces ipomoeae*. Plants were inoculated with *M. enterolobii* (5,000 eggs/plant) and arranged in a completely randomized design in a greenhouse with an average daily temperature of 24.8°C. Galls and egg masses per root system (0-5 scale), eggs per egg mass, eggs per gram of fresh root (gfr), and reproduction factor (RF) were determined. *Meloidogyne enterolobii* infected and reproduced on all the sweetpotato cultivars. The nematode induced galls on both fibrous and storage roots, regardless of the cultivar, as well as induced necrosis and cracks on storage roots. The lesions and cracks on the storage roots were more visually pronounced on Hernandez than those on other cultivars. Cultivar Orleans sustained less root galling and egg masses than other cultivars (*p* ≤ 0.01), and both Orleans and Beauregard cultivars had less eggs per gfr and a lower RF than Covington (5,683 eggs/gfr; RF = 16.92), Evangeline (7,161 eggs/gfr; RF = 30.01), and Hernandez (6,979 eggs/gfr; RF = 22.6). The latter two cultivars sustained the largest amount of reproduction of *M. enterolobii*. The number of eggs per egg mass ranged from 462 to 503 and was similar among all cultivars. In summary, *M. enterolobii* reproduced well on all sweetpotato cultivars; however, differences were observed among cultivars (*p* ≤ 0.001). The host status as previously reported for other root-knot nematode species was not a good predictor of host status to *M. enterolobii*. Some sweetpotato cultivars that were reported as resistant or moderately resistant to *M. incognita* race 3, such as Evangeline and Hernandez, were among the best hosts to *M. enterolobii*. Root growth of Evangeline and Orleans, but not of the other cultivars, was negatively correlated with nematode eggs per gfr.

Sweetpotato (*Ipomoea batatas* (L.) Lam) is a major crop in the southeastern USA and a staple food in many tropical countries around the world. Root-knot nematodes (RKN) (*Meloidogyne* spp.), particularly *M. incognita* (Kofoid and White, 1919) Chitwood, 1949, cause significant suppression in yield and root quality of sweetpotato (Lawrence et al., 1986; Clark et al., 1992). Most recently, there has been an outbreak of *M. enterolobii* (Yang and Eisenback, 1983), Pacara earpod tree RKN (Yang and Eisenback, 1983), observed in the major sweetpotato producing states in the USA. This nematode species was reported causing severe yield and root quality suppression, and in at least one case, total crop loss in North Carolina (Ashby, 2017). Other production states with severe outbreaks of damage include Louisiana (Strain, 2018a, 2018b) and South Carolina (Rutter et al., 2019). In addition, *M. enterolobii* has been reported causing damage on sweetpotato in Africa (Fargette, 1987; Fargette and Braaksma, 1990; Karuki et al., 2017) and China (Gao et al., 2014). The first report of *Meloidogyne enterolobii* in the continental USA occurred in 2001 in Florida, where it was first found infecting ornamental plants and since then numerous other plant species (Brito et al., 2002, 2004, 2008, 2010). Furthermore, in 2013, this nematode species was also found infecting cotton and soybean in North Carolina (Ye et al., 2013). *Meloidogyne enterolobii* is one of the most damaging RKN species noted today because of its wide host range, high degree of virulence, and its ability to overcome RKN-resistant genes in several important agricultural crops, namely *Mi*-1, *Mir1*, *Me*, *N*, *Rk*, and *Tabasco* genes in tomato, soybean, bell pepper, cowpea, and sweet pepper, respectively (Carneiro et al., 2006; Brito et al., 2007; Cetintas et al., 2008; Kiewnick et al., 2009; Pinheiro et al., 2013; Gonçalves et al., 2014; Rosa et al., 2015). Currently, in the USA, this nematode species is of great concern to producers of sweetpotato, cotton, soybean, as well as many other agricultural crops. As a result, regulatory officials in Louisiana, Arkansas, and Mississippi have imposed an external quarantine for *M. enterolobii*. Several sweetpotato cultivars have shown moderate to good resistance to the major *Meloidogyne* spp. including *M. incognita, M. javanica* (Treub, 1885) Chitwood, 1949 and *M. arenaria* (Neal, 1889) Chitwood, 1949 (Cervantes-Flores et al., 2002; Olabiyi, 2007; Marchese et al., 2010). Nevertheless, very little is known about the ability of *M*. *enterolobii* to reproduce on sweetpotatoes in the USA. The objective of this study was to determine the ability of *M. enterolobii* to reproduce on selected sweetpotato cultivars including Beauregard, Covington, Evangeline, Hernandez, and Orleans (LA 05-111). All these cultivars, except Beauregard and Orleans, are reported at least moderately resistant to *M. incognita* race 3 and two other important pathogens to sweetpotato, *Fusarium oxysporum* f. sp. *batatas* and *Streptomyces ipomoeae* (Rolston et al., 1987; LaBonte et al., 1992, 2008, 2012; Yencho et al., 2008).

Nematode isolate, *M. enterolobii* (DPI: N01-00283), was previously identified using both morphological and molecular analyses (Brito et al., 2004). The species was reared on tomato Rutgers in steam-pasteurized soil. Sweetpotato cultivars used in this study were Beauregard, Covington, Evangeline, Hernandez, and Orleans. Tomato Rutgers was used as a control to determine the viability of the inoculum. Eggs were extracted from infected tomato roots using the 0.5% NaOCl (Hussey and Barker, 1973) method as modified by Boneti and Ferraz (1981). An individual sweetpotato stem (20-cm long) from the appropriate cultivar was planted directly into 27-cm-diameter clay pots containing pasteurized soil (89% sand, 3% silt, 5% clay; pH 6.1; 1.1% organic matter). Thirty days after planting the slips, each seedling was inoculated with 5,000 eggs/plant (Pi) at 1,000 eggs/ml in five equal holes 3.5 to 4.5 cm deep surrounding the root system. Plants were set up in a completely randomized design in a greenhouse with 10 replications. The average temperature in the greenhouse was 24.8°C (ITWatchDog, Austin, TX). Plants were watered daily and fertilized once a week with Peter’s fertilizer (20-20-20 with micronutrients) according to the manufacturer’s instructions (United Industries Corp., St. Louis, MO). The experiment was repeated following the same methods and in the same greenhouse.

The tomato and sweetpotato root systems were collected 60 and 90 days after inoculation (DAI), respectively. In both tests, all the sweetpotato cultivars (treatments) were harvested later (90 DAI), when symptoms of nematode infection were clearly visible in storage roots. At harvest, root systems from each experiment were removed from the pots and carefully washed to remove the soil. Storage roots when present were separated from the roots, then root systems were weighted and rated for root galling and egg masses on a 0 to 5 scale, where 0 = 0 galls or egg masses; 1 = 1 to 2 galls or egg masses; 2 = 3 to 10 galls or egg masses, 3 = 11 to 30 galls or egg masses; 4 = 31 to 100 galls or egg masses; and 5 = ≥ 100 galls or egg masses per root system (Taylor and Sasser, 1978). Eggs were extracted from roots as stated above, except that 1% NaOCl was used. Final number of eggs (Pf) for each plant was calculated and eggs per gram of fresh roots (gfr) and the reproductive factor (RF = Pf/Pi) were determined. Host suitability was designated as follows: RF ≥ 1 = good host; 0.1 < RF < 1.0 = poor host; RF ≤ 0.1 = non-host (Sasser et al., 1984). Fecundity was measured by extracting eggs (1% NaOCl) from four egg masses chosen randomly from each plant, totaling 80 egg mass/cultivar.

Statistical analysis was performed on both tests combined, as no significant interaction was observed between cultivars and experiments (minor differences in gall index and egg mass index only) (Table 1). *M. enterolobii* infected and reproduced well on all the sweetpotato cultivars, including those known to have resistance to *M. incognita* race 3. This nematode species caused necrosis and cracks on storage roots (Fig. 1), regardless of the sweetpotato cultivar. Fibrous root fresh weights differed among sweetpotato cultivars being tested for susceptibility to *M. enterolobii,* with Evangeline having the largest root system (22.70 g), whereas Beauregard, Covington, and Orleans cultivars had similar root weights (Table 1). Overall, best nematode hosts were Evangeline and Hernandez followed by Covington, Orleans and Beauregard. Nonetheless, the cultivar Orleans sustained less root galling and egg masses than the other cultivars (*p* ≤ 0.001) (Table 1). Eggs per gram of root were greatest for Evangeline and Hernandez and lowest for Beauregard and Orleans, and total egg production per plant was greatest for Evangeline and lowest for Beauregard and Orleans (Table 1). The number of eggs per egg mass ranged from 462 to 504 and was similar among all cultivars. However, on Orleans no egg masses were found on half of the plants (10 out of 20 plants in both tests). Root growth of Evangeline and Orleans was negatively correlated with *M. enterolobii* root infection (number eggs per gram fresh root), but no such effect was noted for the other cultivars (data not shown). The other cultivars did not show reduced root weight with increasing nematode egg numbers in roots. Sweetpotato is known as a good host to RKN, especially to *M. incognita* and *M. javanica* (Jones and Dukes, 1980; Clark et al., 1992). Our results showed that it is also a good host to *M. enterolobii*. This nematode species has been found reproducing on the sweetpotato cv. CHD in West Africa (Fargette, 1987), and more recently on cultivar Covington in both field (Rutter et al., 2019) and greenhouse conditions (Schwarz et al., 2020) in the USA. All cultivars, except Beauregard, had similar or higher number of eggs per gfr, as compared to tomato (Table 1). Interestingly, sweetpotato is often not a good host to *M. arenaria*, and many cultivars that are highly susceptible to *M. incognita* and *M. javanica*, including Beauregard, have shown good resistance to *M. arenaria* (Cervantes-Flores et al., 2002). Our results indicate that susceptibility to different RKN species can vary widely, and that host status for one RKN species can be very different from other species. Cultivars that showed resistance to *M. incognita* race 3, such as Evangeline and Hernandez (LaBonte et al., 1992, 2008), were susceptible to *M. enterolobii* in this study. Beauregard, which is used to be the predominant sweetpotato cultivar in the USA, has been used as a parental line in breeding programs in many countries, but it is susceptible to two major tropical species of RKN, *M. incognita* and *M. javanica* (Cervantes-Flores et al., 2002) as stated above. In this study, the cultivar Hernandez was susceptible to *M. enterolobii*; however, it has been reported to be resistant to *M. arenaria*, *M. incognita* race 3, and *M. javanica* (Cervantes-Flores et al., 2002); therefore, our findings indicate that the gene (s) that may confer resistance to these nematodes species most likely is not that same as that for *M. enterolobii*, which could make the management of this nematode species more difficult, particularly in areas infested with mixture of species. Covington, which is the predominate cultivar planted in the USA (Barkley et al., 2017), was found to be susceptible to *M. enterolobii* in this study. Likewise, Covington and the NCDM04-001 genotype were reported as susceptible to four North Carolina isolates of *M. enterolobii* (Schwarz et al., 2020). Differential host response of sweetpotato cultivars to different RKN species obscures cultivar selection and nematode management options and emphasizes again the importance of proper nematode identification. Considering the economic importance of the sweetpotato industry to certain states in the USA and our findings, there is a great need to find sources of resistance to *M. enterolobii* and incorporate it into new cultivars. Some progress has been made and some resistance to this nematode species has been identified (Schwarz et al., 2020), which could have significant impacts on the management of this nematode. In summary, all cultivars allowed reproduction of *M. enterolobii*, but at significantly different levels. When using resistance as a tool to manage RKN problems in sweetpotato, cultivar selection should be done based on the RKN species that are present in the field.

**Table 1.** Response of five sweetpotato (*Ipomaea batatas*) cultivars to inoculation with *Meloidogyne enterolobii* in this study.

Factor	Fibrous fresh root weight^u^ (g)	Gall index^v^	Egg mass index^v^	Eggs per egg mass	Eggs per fibrous fresh root weight	Eggs per gram fresh root^x^	RF^w^
*Sweetpotato cultivar*^t^							
Beauregard	15.21 bc	4.65 a	3.65 b	475 a	30,975 c	2,668 c	6.20 c
Covington	14.99 bc	4.25 a	4.45 ab	490 a	84,585 b	5,683 ab	16.92 b
Evangeline	22.70 a	4.65 a	4.80 a	503 a	150,070 a	7,161 a	30.01 a
Hernandez	16.35 b	4.70 a	4.90 a	462 a	113,235 b	6,979 a	22.65 b
Orleans	11.65 c	1.95 b	2.30 c	475 a	42,022 c	3,678 bc	8.41 c
*Test*							
Test 1	15.42	4.24	4.22	504	81,325	5,444	16.27
Test 2	16.94	3.84	3.82	459	87,030	5,024	17.41
*F* probability cv	< 0.001	< 0.001	< 0.001	> 0.50	< 0.001	< 0.001	< 0.001
*F* probability test	0.12	0.06	0.10	> 0.50	0.46	0.50	0.46
Tomato	37.7	5	5	n/a^x^	176,340	4,801	35.27

**Notes:** ^t^Inoculum level: 5,000 eggs/plant; ^u^Means are average of duplicate tests, each with 10 and 5 replicates for all sweetpotato cultivars and tomato, respectively. Means followed by a common letter are not different according to Duncan’s multiple-range test (*p* ≤ 0.001); ^v^Gall and egg mass index: 0 = 0 galls or egg masses, 1 = 1 to 2 galls or egg masses, 2 = 3 to 10 galls or egg masses, 3 = 11 to 30 galls or egg masses, 4 = 31 to 100 galls or egg masses, and 5 = ≥ 100 galls or egg masses per root system (Taylor and Sasser, 1978); ^w^RF = reproduction factor (Sasser et al., 1984); ^x^n/a = Not applicable.

**Figure 1:**
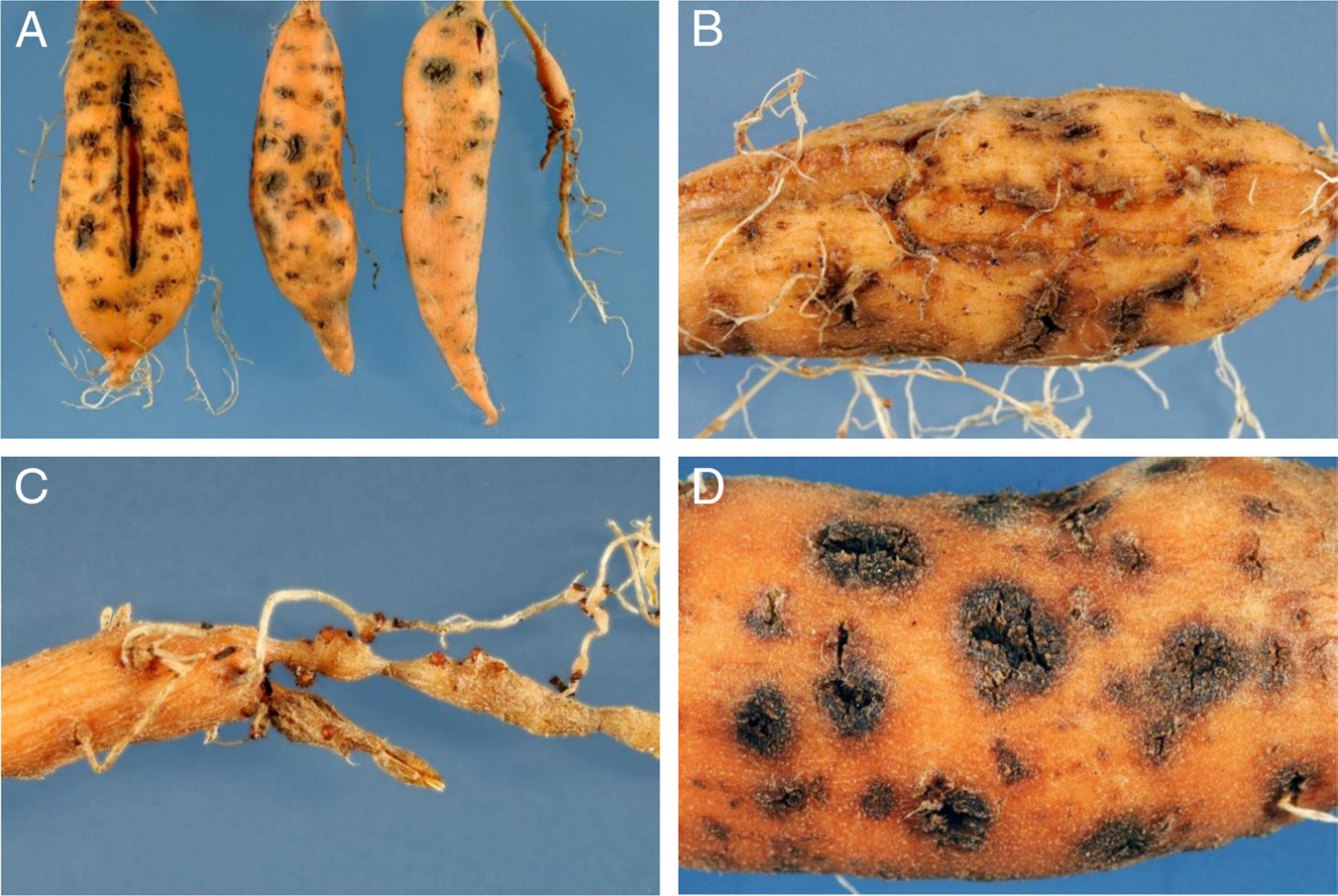
Sweetpotato cv. Hernandez infected with *Meloidogyne enterolobii*. (A) Symptoms induced in the storage roots 90 days after nematode inoculation; (B) Necrosis and cracks of storage roots, induced by *M. enterolobii*; also, noticeable galls and egg masses on fibrous roots; (C) A close up of the galls and large egg masses on both fibrous and storage roots; and (D) Close up of necrosis and cracks in storage roots.
